# DAPTA, a C-C Chemokine Receptor 5 (CCR5), Leads to the Downregulation of Notch/NF-κB Signaling and Proinflammatory Mediators in CD40^+^ Cells in Experimental Autoimmune Encephalomyelitis Model in SJL/J Mice

**DOI:** 10.3390/biomedicines11061511

**Published:** 2023-05-23

**Authors:** Hanan Alghibiwi, Mushtaq A. Ansari, Ahmed Nadeem, Majed Ali Algonaiah, Sabry M. Attia, Saleh A. Bakheet, Thamer H. Albekairi, Sultan Almudimeegh, Abdullah S. Alhamed, Mudassar Shahid, Mohammad Y. Alwetaid, Yasseen A. Alassmrry, Sheikh F. Ahmad

**Affiliations:** 1 Department of Pharmacology and Toxicology, College of Pharmacy, King Saud University, Riyadh 11451, Saudi Arabia; 2 Department of Pharmaceutics, College of Pharmacy, King Saud University, Riyadh 11451, Saudi Arabia; 3 Department of Botany and Microbiology, College of Science, King Saud University, Riyadh 11451, Saudi Arabia

**Keywords:** CCR5 antagonist, Notch signaling, NF-κB/IκB-α, B lymphocytes, EAE, multiple sclerosis

## Abstract

Multiple sclerosis (MS) is an autoimmune inflammatory disease of the central nervous system characterized by motor deficits, cognitive impairment, fatigue, pain, and sensory and visual dysfunction. CD40, highly expressed in B cells, plays a significant role in MS pathogenesis. The experimental autoimmune encephalomyelitis (EAE) mouse model of MS has been well established, as well as its relevance in MS patients. This study aimed to evaluate the therapeutic potential of DAPTA, a selective C-C chemokine receptor 5 (CCR5) antagonist in the murine model of MS, and to expand the knowledge of its mechanism of action. Following the induction of EAE, DAPTA was administrated (0.01 mg/kg, i.p.) daily from day 14 to day 42. We investigated the effects of DAPTA on NF-κB p65, IκBα, Notch-1, Notch-3, GM-CSF, MCP-1, iNOS, and TNF-α in CD40^+^ spleen B cells using flow cytometry. Furthermore, we also analyzed the effect of DAPTA on NF-κB p65, IκBα, Notch-1, Notch-3, GM-CSF, MCP-1, iNOS, and TNF-α mRNA expression levels using qRT-PCR in brain tissue. EAE mice treated with DAPTA showed substantial reductions in NF-κB p65, Notch-1, Notch-3, GM-CSF, MCP-1, iNOS, and TNF-α but an increase in the IκBα of CD40^+^ B lymphocytes. Moreover, EAE mice treated with DAPTA displayed decreased NF-κB p65, Notch-1, Notch-3, GM-CSF, MCP-1, iNOS, and TNF-α and but showed increased IκBα mRNA expression levels. This study showed that DAPTA has significant neuroprotective potential in EAE via the downregulation of inflammatory mediators and NF-κB/Notch signaling. Collectively, DAPTA might have potential therapeutic targets for use in MS treatment.

## 1. Introduction

Multiple sclerosis (MS) is an inflammatory disease affecting the central nervous system (CNS) [1,2]. The clinical features of MS are diverse and mainly include limb weakness, blurred vision, ataxia, paresthesia, fatigue, and cognitive deficits [3]. In addition, there are progressive neuronal and oligodendroglial losses in MS [4]. EAE mimics many of the critical physiopathological features of MS in humans [5]. The etiology of MS is still unknown, but converging lines of evidence suggest that it is a multifactorial disease of complex tract caused by a dysregulation of the immune system [6]. The role of B cells has recently become a central issue in MS. B cells’ involvement in MS has primarily been considered in terms of the potential role of pathogenic CNS-reactive antibodies [7,8]. To date, immunosuppressive and immunomodulatory medications are the major therapeutic strategies for the treatment of MS, but they carry severe side effects [9]. None of the drugs have proven helpful in treating MS. Due to the high prevalence of MS patients worldwide and poor therapeutic outcomes, most MS patients eventually develop a disability [10,11]. Hence, seeking an effective therapeutic strategy for MS treatment is timely and essential.

The transcription factor NF-κB activation is considered to be a major source of inflammatory cascades in several neuroinflammatory disorders that exacerbate the disease pathogenesis in various CNS diseases, including autoimmune encephalomyelitis [12]. NF-κB activation occurs in microglia, astrocytes, oligodendrocytes, macrophages, and neurons, initiating initiate inflammatory reactions in EAE [13]. Previous results also indicated that NF-κB activation in inflammatory cells enhances inflammation and facilitates MS and EAE development [14]. It has been reported that CD40-mediated NF-κB activation in B cells is increased in MS [15]. Several studies have focused on evaluating Notch signaling during EAE [16,17]. Notch signaling has been implicated as a significant contributor to remyelination failure in MS [16]. Previous results showed that the Notch pathway plays a vital role in controlling inflammatory reactions in the CNS [18].

GM-CSF has been demonstrated to be critical for EAE pathogenesis due to the role it plays in driving the mobilization and activation of neutrophils [19]. A previous study has demonstrated that GM-CSF blockade in the relapsing-remitting (RR)-EAE model prevented disease relapses [20]. It has been previously demonstrated that GM-CSF activates microglia within the CNS and recruits and stimulates peripheral macrophages and dendritic cells during EAE [21]. It has been reported that GM-CSF promotes chronic disability in EAE by altering the composition of CNS myeloid cells [22]. Several studies have described the expression of MCP1 in the CNS of patients with MS using autopsy tissue [23,24]. Previous results from the use of reactive astrocytes also suggested a significant role of MCP-1 in demyelinating MS lesions [25]. MCP-1 was also identified within hypertrophic astrocytes in a noninflamed submeningeal region adjacent to chronic active lesions [26].

TNF-α plays a diverse role in autoimmune and inflammatory disorders [27]. A previous study highlighted that TNF-α increases brain-derived neurotrophic factor expression in trigeminal ganglion neurons [28]. A previous study also indicated that TNF-α is elevated in MS patients and correlated with disease progression [29]. TNF-α is also upregulated in demyelinating lesions in EAE in order to promote neuronal excitotoxicity and oligodendrocyte death [30]. A previous study exhibited that the inducible nitric oxide synthase (iNOS) expression significantly increased in the spinal cords of EAE mice [31]. It has also been reported that the activated microglia revealed iNOS-positive immunoreactions in the perivascular regions of spinal cords in animals with EAE [32]. Additionally, the suppression of iNOS is associated with the amelioration of EAE [33].

Chemokines and their receptors are expressed in endothelial cells, microglia, astrocytes, and neurons [34]. Previous studies reported the pathogenic role of chemokine receptors in EAE [35,36]. The levels of EAE induced in CCR5-knockout mice reveal a decrease in infiltrating immune cells, indicating the important role of CCR5 in cell recruitment into the CNS [37]. Moreover, CCR5 is considered important for leukocyte extravasation, thus facilitating the migration of these cells into the CNS [38]. Peripheral blood CCR5^+^ cells secrete high levels of IFN-γ, and IFN-γ is observed in demyelinating lesions [39]. The frequency of CCR5^+^ cells is elevated in EAE and MS [39,40]. Chemokine receptor antagonists ameliorate EAE progression [41]. D-ala-peptide T-amide (DAPTA) is a selective CCR5 antagonist [42]. It has been shown that the administration of DAPTA reduces microglia and astrocyte activation within the hippocampus [43]. In addition, DAPTA treatment appears to be a therapeutic target for the treatment of treating inflammation-related diseases [44]. Our previous study demonstrated that DAPTA administration attenuates immune aberrations by downregulating Th9/Th17 immune responses in the BTBR mouse model of autism [45]. Recently, we also demonstrated that DAPTA treatment alleviates inflammation by regulating IFN-γ/IL-10 and STAT4/Smad3 signaling in a mouse model of EAE [46].

EAE, the most widely used experimental model for MS, is a universal animal model resembling human RR-MS [47]. This model has been extensively used to discover new therapies for application to MS. Recently, we reported that treatment with DAPTA exerts an excellent therapeutic effect in the EAE mouse model [46]. Therefore, we hypothesized that the administration of DAPTA could downregulate proinflammatory mediators and Notch/NF-κB signaling in B cells in the SJL/J mouse model of RR-MS. In this study, we explore the possible molecular mechanisms underlying these effects.

## 2. Materials and Methods

### 2.1. Chemicals

DAPTA was purchased from Tocris Bioscience (Bristol, UK). Fluorescein isothiocyanate, PE/Cyanine7, Allophycocyanin, Alexa Fluor^®^ 488, PE/Dazzle™ 594, Alexa Fluor^®^ 647, and phycoerythrin-labeled CD40, NF-κB p65, IκBα, Notch1, Notch3, GM-CSF, iNOS, RORγT, MCP-1, TNF-α, lysis, and permeabilization solution, and fixation buffers were purchased from BD Biosciences and BioLegend (San Diego, CA, USA). SYBR Green and cDNA kits were purchased from Applied Biosystems (Foster City, CA, USA). Primers were purchased from GenScript (Piscataway, NJ, USA). TRizol was purchased from Invitrogen (Carlsbad, CA, USA).

### 2.2. Animals

The Bioethics Committee of the College of Pharmacy, King Saud University, approved all experimental procedures. Female SJL/J mice, aged 7–8 weeks, were purchased from the Jackson Laboratories (Bar Harbor, ME, USA). The mice were housed in specific pathogen-free conditions in an appropriate environment, maintained in a 12 h light/dark cycle, and given ad libitum access to food and water. Mice were allowed to acclimatize for two weeks before the initiation of the experiment. 

### 2.3. EAE Induction and DAPTA Treatment

In order to establish the EAE model, SJL/J mice were immunized subcutaneously with 200 µg of proteolipid protein (PLP139-151) peptide (Hooke Laboratories, Lawrence, MA, USA). Each mouse received an additional 200 ng of pertussis toxin (PTX) intraperitoneally (i.p.) on the immunization day [46,48]. DAPTA injection was started from day 14 after immunization and continued once daily until day 42 at a dose of DAPTA (0.01 mg/kg, i.p.). These parameters were selected based on the previous studies [43,44,45,46,47]. The mice in the standard control and EAE model groups were injected daily with the same volume of saline. Mice were sacrificed at the end of the treatment period by deep inhalational anesthesia (isoflurane). Different tissues (brain/spleen) were collected for use in various molecular analyses, i.e., flow cytometry and RT-PCR.

### 2.4. Flow Cytometry Analysis

The following conjugated monoclonal antibodies were used for the flow cytometry analysis of spleen cells: anti-CD40 PE/Dazzle, anti-CD40 APC, anti-CD40 FITC anti-NF-κB p65 FITC, anti-IκBα PE/Cyanine7, anti-Notch1 PE/Cyanine7, anti-Notch3 APC, anti-GM-CSF Alexa Fluor^®^ 488, anti-iNOS PE/Dazzle, anti-MCP-1 APC, and anti-TNF-α PE/Cyanine7. Cells were washed, and surface staining of CD40 was performed. Intracellular staining was performed after fixing and permeabilizing for anti-NF-κB p65, anti-IκBα, anti-Notch1, anti-Notch3, anti-GM-CSF, anti-iNOS, anti-MCP-1, and anti-TNF-α fluorescent antibodies. The proportions of CD40^+^NF-κB p65^+^, CD40^+^IκBα^+^, CD40^+^Notch1^+^, CD40^+^Notch3^+^, CD40^+^GM-CSF^+^, CD40^+^iNOS^+^, CD40^+^MCP-1^+^, and CD40^+^TNF-α^+^ cells were determined in the lymphocyte gate. Sample analysis was performed with FACS (FC500), and data analysis was conducted using CXP software (Beckman CoulterIndianapolis, Indianapolis, IN, USA) [46].

### 2.5. Real-Time PCR Analysis

According to the manufacturer’s protocol, RNA from the brain was extracted using a TRIzol reagent (Invitrogen, Carlsbad, CA, USA). NanoDrop 2000 measured the concentration of RNA. cDNA was prepared using a cDNA reverse transcription kit, followed by RT-PCR with SYBR Green PCR reagents (Applied Biosystems, Foster City, CA, USA). The primer sequences used for the assay were as follows: *Nf-κB p65*, forward 5′-CTGCCGAGTAAACCGGAACT-3′, reverse 5′-CCCTGTGACATCACCTGCTT-3′; *IκBα*, forward 5′-AAGGCTACTCCCCCTACCAG-3′, reverse 5′-CAAGAAGGCGACACAGACCT-3′; *Notch1*, forward 5′-GGTGCTCTGATGGACGACAA-3′, reverse 5′-CATGAGGGGTGTGAAGCCAT-3′; *Notch3*, forward 5′-AGGCCATGGTCTTCCCCTAT-3′, reverse 5′-ACCTCCCCCATCAGACTCTC-3′; *Gm-csf*, forward 5′-AGAGGCCATCAAAGAAGCCC-3′, reverse 5′AAATTGCCCCGTAGACCCTG-3′; *Mcp-1*, forward 5′-GATTCTCCGGCCCATGAGAG-3′, reverse 5′-AAGGATGTTCTTCCCAGCGG-3′; *Inos*, forward 5′-GTGACCATGGAGCATCCCAA-3′, reverse 5′-CGATGTCATGAGCAAAGGCG-3′; *Tnf-α*, forward 5′-TGTCTACTCCTCAGAGCCCC-3′, reverse 5′-GGGGGAGAGGTAGGGATGTT-3′; and *Gapdh*, forward 5′-TGTGAACGGATTTGGCCGTA-3′, reverse 5′-ACTGTGCCGTTGAATTTGCC-3′. The samples were normalized to the internal control *Gapdh*, and relative expression was calculated using the 2^−ΔΔCT^ method [49].

### 2.6. Statistical Analysis

The raw data were first tested for homogeneity (F and Bartlett’s test) and normality (Kolmogorov–Smirnov test) of variance and were found to be normally distributed and homogenous. Thus, statistical analysis was performed with parametric tests without data transformation. Data are presented as the mean ±SD. One-way analysis of variance (ANOVA) followed by Tukey’s multiple comparisons post hoc test or Student’s *t* test was performed to determine the difference between the two groups where appropriate. Statistical analysis was performed using GraphPad-Prism 5.0 (GraphPad Software, San Diego, CA, USA). A *p* value below 0.05 was considered statistically significant.

## 3. Results

### 3.1. DAPTA Treatment Regulates NF-κB p65/IκBα Expression in EAE Mice

**Figure 1:**
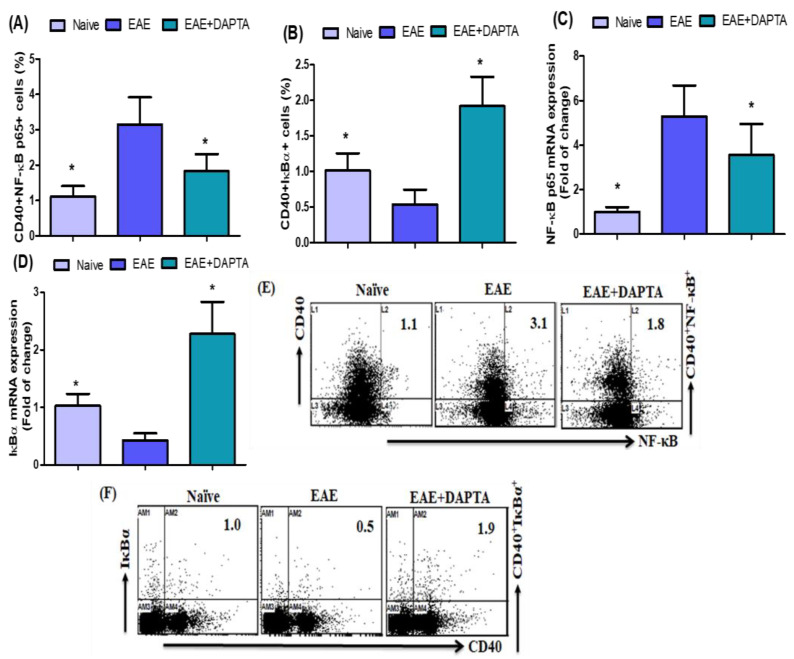
(**A**,**B**) Effects of DAPTA on NF-κB p65- and IκBα-expressing CD40^+^ B cells in the spleen cells were analyzed using flow cytometry. (**C**,**D**) Effects of DAPTA on *NF-κB p65* and *IκBα* mRNA expression levels were analyzed using RT-PCR in brain tissue. (**E**,**F**) Flow cytometry representative dot plots of CD40^+^NF-κB p65^+^ and CD40^+^IκBα^+^ expressions are shown in one mouse from each group. Naive SJL/J mice were treated with saline (naive), EAE mice were treated with saline (EAE), and EAE mice were treated with DAPTA (EAE+DAPTA) at 0.01 mg/kg, i.p., once daily from day 14 to day 42. All data are shown as mean ± SD (*n* = 6). * *p* < 0.05.

In order to investigate the effect of DAPTA on NF-κB p65- and IκBα-expressing CD40^+^ B cells, we performed flow cytometric analysis of splenic cells from untreated and DAPTA-treated EAE mice (Figure 1A,B) to confirm whether DAPTA affects *Nf-κB p65* and *IκBα* mRNA expression in the brain tissue. Our results showed that NF-κB p65-expressing CD40^+^ B cells were significantly decreased in DAPTA-treated EAE mice, whereas the presence of IκBα-expressing CD40^+^ B cells increased greatly compared to the levels displayed in untreated EAE mice (Figure 1A,B). Our results showed that *NF-κB p65* mRNA expression was decreased in DAPTA-treated EAE mice, whereas *IκBα* expression was increased compared to untreated EAE mice (Figure 1C,D). Our results demonstrated that DAPTA treatment attenuates immune imbalances, a function is likely performed by regulating NF-κB/IκBα activation.

### 3.2. Effect of DAPTA on Notch1 and Notch3 Signaling

**Figure 2:**
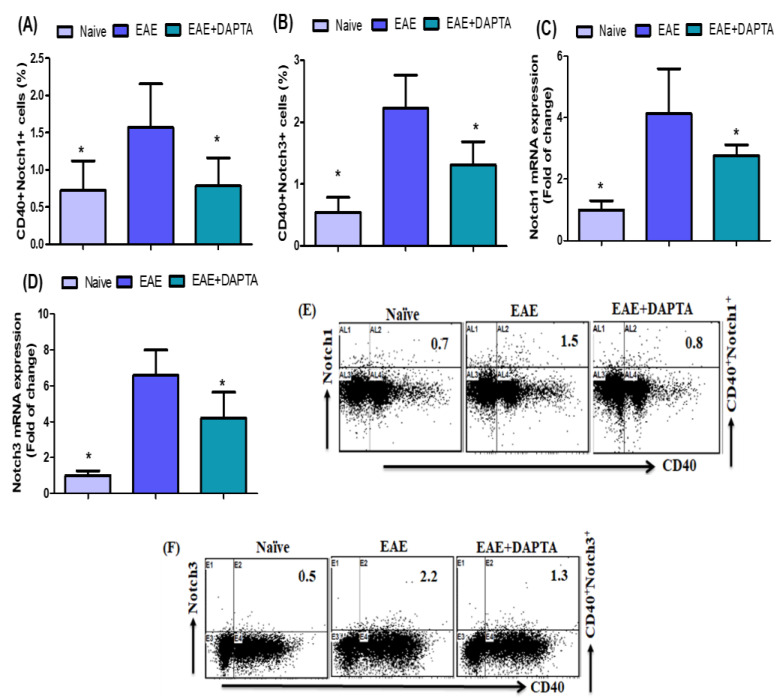
(**A**,**B**) Effects of DAPTA on Notch1- and Notch3-expressing CD40^+^ B cells in the spleen cells were analyzed using flow cytometry. (**C**,**D**) Effects of DAPTA on *Notch1* and *Notch3* mRNA expression levels were analyzed using RT-PCR in brain tissue. (**E**,**F**) Flow cytometry representative dot plots of CD40^+^Notch1^+^ and CD40^+^ Notch3^+^ expressions are shown in one mouse from each group. Naive SJL/J mice were treated with saline (naive), EAE mice were treated with saline (EAE), and EAE mice were treated with DAPTA (EAE+DAPTA), at 0.01 mg/kg, i.p., once daily from day 14 to day 42. All data are shown as mean ± SD (*n* = 6). * *p* < 0.05.

To further investigate the effect of DAPTA on EAE mice, flow cytometric analysis was performed to evaluate Notch1- and Notch3-expressing CD40^+^ B cells in the spleen. Our results revealed that EAE mice treated with DAPTA harbored fewer Notch1- and Notch3-expressing CD40^+^ B cells than untreated EAE mice (Figure 2A,B). We further investigated the mRNA expression levels of the potent inflammatory signaling *Notch1* and *Notch3* in the brain tissue. We observed a significant reduction in *Notch1* and *Notch3* mRNA expression levels in DAPTA-treated EAE mice compared to the levels exhibited in untreated EAE mice (Figure 2C,D). These results suggested that DAPTA administration can restore immune abnormalities during MS.

### 3.3. DAPTA Downregulates GM-CSF and MCP-1 Expression in EAE Mice

**Figure 3:**
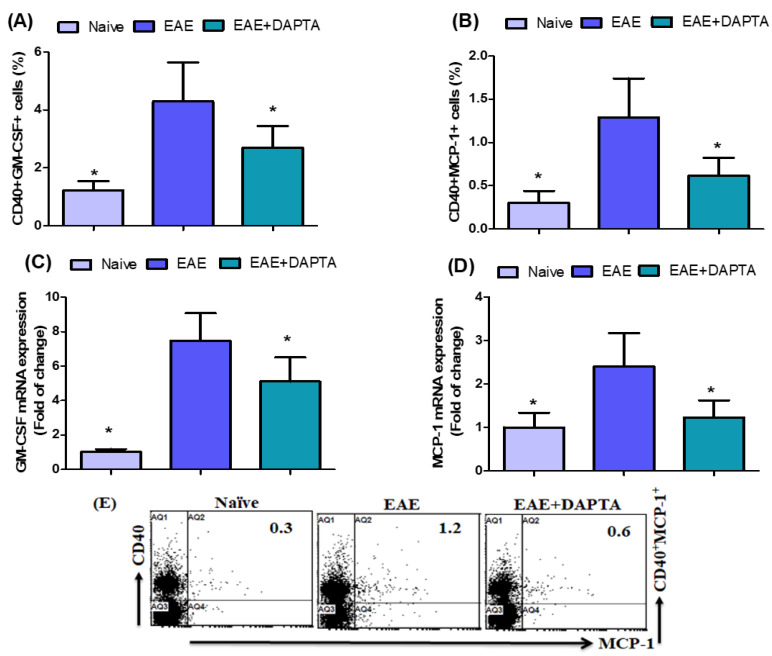
(**A**,**B**) Effects of DAPTA on GM-CSF- and MCP-1-expressing CD40^+^ B cells in the spleen cells were analyzed using flow cytometry. (**C**,**D**) Effects of DAPTA on *Gm-csf* and *Mcp-1* mRNA expression levels were analyzed using RT-PCR in brain tissue. (**E**) Flow cytometry representative dot plots of CD40^+^MCP-1^+^ expression are shown in one mouse from each group. Naive SJL/J mice were treated with saline (naive), EAE mice were treated with saline (EAE), and EAE mice were treated with DAPTA (EAE+DAPTA), 0.01 mg/kg, i.p., once daily from day 14 to day 42. All data are shown as mean ± SD (*n* = 6). * *p* < 0.05.

We further revealed the effect of DAPTA on GM-CSF- and MCP-1-expressing CD40^+^ B cells in the spleen. We showed that the proportion of GM-CSF- and MCP-1-expressing CD40^+^ B cells decreased in EAE mice treated with DAPTA compared to that visible in untreated EAE mice (Figure 3A,B). To further explain the mechanism of action of DAPTA, we examined changes in *Gm-csf* and *Mcp-1* mRNA expression levels in brain tissue. DAPTA treatment in EAE mice caused a significant decrease in *Gm-csf* and *Mcp-1* at the mRNA levels compared to untreated EAE mice (Figure 3C,D). These data suggest that DAPTA administration may be anti-inflammatory in the EAE mouse model.

### 3.4. DAPTA Downregulates Proinflammatory Mediators in EAE Mice

**Figure 4:**
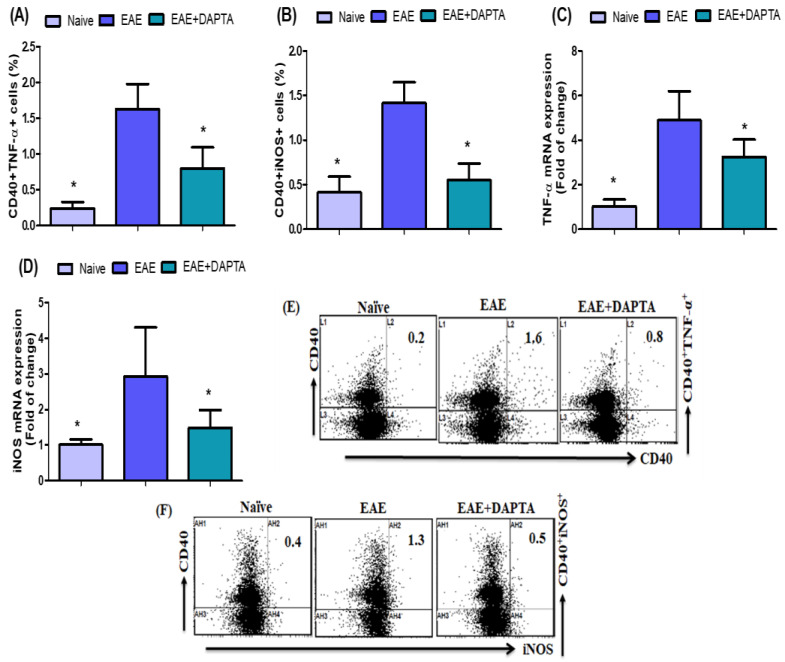
(**A**,**B**) Effects of DAPTA on TNF-α-and iNOS-expressing CD40^+^ B cells in the spleen cells were analyzed using flow cytometry. (**C**,**D**) Effects of DAPTA on *Tnf-α* and *Inos* mRNA expression levels were analyzed using RT-PCR in brain tissue. (**E**,**F**) Flow cytometry representative dot plots of CD40^+^TNF-α^+^ and CD40^+^iNOS^+^ expressions are shown in one mouse from each group. Naive SJL/J mice were treated with saline (naive), EAE mice were treated with saline (EAE), and EAE mice were treated with DAPTA (EAE+DAPTA), 0.01 mg/kg, i.p., once daily from day 14 to day 42. All data are shown as mean ± SD (*n* = 6). * *p* < 0.05.

We further evaluated the number of TNF-α- and iNOS-expressing CD40^+^ B cells in the spleen. We found a significant increase in TNF-α- and iNOS-expressing CD40^+^ B cells in untreated EAE mice. Our data indicate that DAPTA treatment in EAE mice significantly decreased TNF-α- and iNOS-expressing CD40^+^ B cells compared to untreated EAE mice (Figure 4A,B). We also measured the mRNA expression to confirm decreased *Tnf-α* and *Inos* mRNA levels in the brain tissue. DAPTA-treated EAE mice showed a significant reduction in *Tnf-α* and *Inos* mRNA levels compared to the EAE untreated mice (Figure 4C,D). These results prove that DAPTA of EAE has been involved in the anti-inflammatory response and suppressed the expressions of both TNF-α and iNOS.

## 4. Discussion

MS is a chronic autoimmune inflammatory disease of the CNS. MS is observed to result from an intricate interplay between inflammatory effector cells [50,51]. The animal model for exploring neuroinflammation is EAE, which shares significant characteristics with MS [52]. B cells play an essential role in the pathogenesis of MS. [53]. B cell repertory in the CNS and the periphery are closely associated, suggesting that disease-relevant B cell networks interact at both sides of the blood–brain barrier [54,55]. There is evidence that the B cells of MS patients exhibited aberrant proinflammatory cytokine responses [56]. In MS, CD40 is expressed in different cell types, including B cells, macrophages, endothelial cells, and resident CNS [57]. Previously, it has been demonstrated that CD40 plays a key role during neuroinflammation progression [58]. The CD40 signaling pathway has been well documented as an immune checkpoint and humoral and cellular immunity stimulator. The CD40 signaling pathway in APCs contributes to numerous cellular functions, such as Th17 cell polarization, proinflammatory cytokine release, and immunoglobulin isotype switching [59]. Furthermore, CD40 deficiency inhibits leukocyte infiltration into the CNS [60]. Therefore, this study explored the overall inflammatory potential of APCs expressing CD40 in EAE mice.

NF-κB is the major transcription factor that regulates inflammation in inflammatory diseases, including MS and EAE [14,61]. The NF-κB signaling cascade plays a vital role in the modulation of immune and inflammatory responses, which has been associated with developing autoimmune demyelinating diseases [12]. Many studies have found that NF-κB is activated in the brain tissue of patients with MS [13]. Previous studies suggested that NF-κB activation promotes oligodendrocyte survival during inflammatory insults [62]; some findings determined that NF-κB plays a critical role in the resident cells of the CNS during disease progression [63,64]. The current study aimed to evaluate the therapeutic potential and underlying mechanisms of CCR5 antagonist in an animal model of MS. We demonstrated that EAE mice, treated with DAPTA, significantly exhibited decreased CD40^+^NF-κB p65^+^ and increased CD40^+^IκBα^+^ cells in the spleen. RT-PCR was used to analyze the molecular mechanisms related to inflammation in the brain. Therefore, we found that EAE mice treated with DAPTA downregulated *Nf-κB p65* and upregulated *IκBα* mRNA expression in brain tissue. This study showed that DAPTA has significant neuroprotective potential in EAE via modulation of NF-κB/IκBα signaling. Therefore, we supposed that DAPTA might effectively treat or improve MS.

Notch signaling plays multiple roles in health and disease [65]. Notch signaling is an essential regulator of type 2 immunity [66]. A recent report indicated that Notch signaling regulates T-cell accumulation and function in the CNS during EAE [67]. In addition, substantial evidence showed that inhibition of Notch signaling ameliorates the clinical course of EAE [17]. Existing evidence suggests that the absence of Notch expression in murine myeloid cells attenuates the development of EAE [68]. Another study also showed that the inhibition of Notch signaling attenuates the development of EAE [69]. The aims of this study were to further evaluate the cellular and molecular mechanisms of CCR5 antagonists in the EAE mouse model. In our present study, DAPTA treatment decreased Notch1- and Notch3-expressing CD40^+^ B cells in the spleen of EAE mice. In addition, the mRNA expression levels of *Notch1* and *Notch3* were significantly decreased by DAPTA treatment in EAE brain tissue, playing a crucial role in preventing MS/EAE. These results support our hypothesis that DAPTA reduces Notch1/Notch3 production/expression in EAE mice. Our data suggest that decreases in Notch1/Notch3 signaling in EAE mice could be one of the mechanisms by which DAPTA attenuates MS progression.

The critical role of GM-CSF in EAE and MS was recently reported [70]. The expression of GM-CSF is significantly increased in MS [71]. A previous study has shown that GM-CSF is critical in MS pathogenesis and EAE development since GM-CSF-deficient mice resist EAE induction [72]. GM-CSF signaling was shown to be sufficient to initiate EAE and was produced by myeloid cells infiltrating the CNS sustained neuroinflammation [73]. GM-CSF promotes chronic disability in EAE patients by altering the composition of CNS-infiltrating cells [22]. MCP-1 expression correlated with clinical severity and attacks in relapsing EAE [74]. MCP-1 expression was followed by further infiltration of monocytes into the CNS and the onset of clinical signs [75]. Infiltrating neutrophils, macrophages, and CD8 T cells can produce MCP1 in EAE [76]. CCL2 expression was upregulated in gamma interferon knockout (GKO) mice with EAE [77]. The use of anti-MCP1 using DNA vaccination or antibodies during relapsing EAE reduced the severity of the disease [74,75,76,77,78]. We further explored the therapeutic role of the CCR5 antagonist DAPTA in EAE mice. In this study, we showed that GM-CSF- and MCP-1-expressing CD40^+^ B cells were significantly decreased in mice with EAE by the treatment of DAPTA. We further observed that Gm-csf and Mcp-1 mRNA expression levels were considerably reduced in DAPTA-treated mice with EAE. DAPTA may represent a new target for inhibiting inflammatory processes in MS development. Therefore, these results support our hypotheses that a CCR5 antagonist could be a potential therapeutic target for treating neuroimmune disorders, including MS.

iNOS is expressed in microglial cells, astrocytes, and neurons in the CNS [79]. It has been shown that there are increased levels of iNOS protein in the CNS in MS [80]. A previous study also observed that iNOS expression was raised in the EAE mice brain [81]. A clinical association between iNOS and pathogenesis has been reported in MS and EAE [82,83]. During chronic demyelination, a pathogenic phenotype of microglial cells is associated with iNOS expression [84]. A previous study showed TNF-α expression by resident microglia and infiltrating leukocytes in the CNS of mice with EAE [85]. In the present study, it has been revealed that EAE mice treated with DAPTA downregulate proinflammatory mediators, namely, iNOS- and TNF-α-expressing CD40^+^ B cells. DAPTA treatment inEAE mice also caused a reduction in the *Inos* and *Tnf-α* mRNA expression levels. This study has demonstrated that DAPTA treatment may downregulate the expression of proinflammatory mediators in the spleen and brain of the EAE mice, eventually attenuating EAE development. These observations suggested that DAPTA may potentially alleviate MS. Therefore, these findings may lead to the development of a new drug for treating neurodegenerative diseases, including MS. Further research is warranted to explore the complex molecular pathways of DAPTA in the EAE model.

## 5. Conclusions

Our results demonstrate the critical role played by inflammatory mediators and NF-κB/Notch signaling in RR-EAE and provide a novel therapeutic approach. CCR5 antagonist leads to a significant reduction in NF-κB/Notch signaling in EAE mice. Additionally, CCR5 antagonist reduces inflammatory mediators such as GM-CSF/iNOS and MCP-1/TNF-α in EAE; this points to the potential benefits of CCR5 antagonist as a novel therapeutic target for the treatment of MS. Therefore, these findings provide additional evidence that DAPTA has possible effects on the treatment of MS and expand our knowledge of the mechanism of action of DAPTA in the EAE model.

## Data Availability

All data presented in this study are available on reasonable request from the corresponding author.

## Abbreviations

Multiple sclerosis (MS); experimental autoimmune encephalomyelitis (EAE); C-C chemokine receptor 5 (CCR5); central nervous system (CNS); inducible nitric oxide synthase (iNOS); D-Ala-peptide T-amide (DAPTA); proteolipid protein (PLP); complementary DNA (cDNA); nuclear factor kappa-light-chain-enhancer of activated B cells (NF-κB); granulocyte-macrophage colony-stimulating factor (GM-CSF); tumor necrosis factor alpha (TNF-α); monocyte chemoattractant protein-1 (MCP-1); intraperitoneally (i.p.); phycoerythrin (PE); allophycocyanin (APC); fluoroisothiocyanate (FITC); glyceraldehyde 3-phosphate dehydrogenase (GAPDH); messenger RNA (mRNA); reverse transcription polymerase chain reaction (RT-PCR). Roswell Park Memorial Institute (RPMI) 1640; Proteolipid protein (PLP).
